# Morphological characterization and transcriptome analysis of leaf angle mutant *bhlh112* in maize [*Zea mays* L.]

**DOI:** 10.3389/fpls.2022.995815

**Published:** 2022-10-07

**Authors:** Yunfang Zhang, Xiangzhuo Ji, Jinhong Xian, Yinxia Wang, Yunling Peng

**Affiliations:** ^1^ College of Agronomy, Gansu Agricultural University, Lanzhou, China; ^2^ Gansu Provincial Key Laboratory of Aridland Crop Science, Gansu Agricultural University, Lanzhou, China; ^3^ Gansu Key Laboratory of Crop Improvement & Germplasm Enhancement, Gansu Agricultural University, Lanzhou, China

**Keywords:** maize (Zea mays L.), *bhlh112* mutant, transcriptomics, exogenous hormones, co-expression network

## Abstract

Leaf angle is an important agronomic trait in maize [*Zea mays* L.]. The compact plant phenotype, with a smaller leaf angle, is suited for high-density planting and thus for increasing crop yields. Here, we studied the ethyl methane sulfonate (EMS)-induced mutant *bhlh112*. Leaf angle and plant height were significantly decreased in *bhlh112* compared to the wild-type plants. After treatment of seedlings with exogenous IAA and ABA respectively, under the optimal concentration of exogenous hormones, the variation of leaf angle of the mutant was more obvious than that of the wild-type, which indicated that the mutant was more sensitive to exogenous hormones. Transcriptome analysis showed that the *ZmbHLH112* gene was related to the biosynthesis of auxin and brassinosteroids, and involved in the activation of genes related to the auxin and brassinosteroid signal pathways as well as cell elongation. Among the GO enrichment terms, we found many differentially expressed genes (DEGs) enriched in the cell membrane and ribosomal biosynthesis, hormone biosynthesis and signaling pathways, and flavonoid biosynthesis, which could influence cell growth and the level of endogenous hormones affecting leaf angle. Therefore, *ZmbHLH112* might regulate leaf angle development through the auxin signaling and the brassinosteroid biosynthesis pathways. 12 genes related to the development of leaf were screened by WGCNA; In GO enrichment and KEGG pathways, the genes were mainly enriched in rRNA binding, ribosome biogenesis, Structural constituent of ribosome; *Arabidopsis* ribosome RNA methyltransferase CMAL is involved in plant development, likely by modulating auxin derived signaling pathways; The free 60s ribosomes and polysomes in the functional defective mutant *rice minute-like1* (*rml1*) were significantly reduced, resulting in plant phenotypic diminution, narrow leaves, and growth retardation; Hence, ribosomal subunits may play an important role in leaf development. These results provide a foundation for further elucidation of the molecular mechanism of the regulation of leaf angle in maize.

## 1 Introduction

Maize (*Zea mays* L.) is one of the three largest food crops grown in China (Yang et al., 2019). It is also a common feed for animals and the raw material for various industrial products (He et al., 2020). Since 2012, maize has become the most produced food crop in the world, and together with wheat and rice, it plays an important role in ensuring national food security and meeting market demand (Zhao et al., 2016).

Plant height and leaf angle are important plant architecture traits: plant height is closely related to lodging resistance, and leaf angle is closely associated with canopy structure and photosynthetic efficiency under high planting density. It is important to improve plant lodging resistance and increase canopy photosynthetic area when planting in high densities to improve yield. Previous research has shown that phytohormones, such as auxin indole-3-acetic acid (IAA), gibberellins (GAs), and brassinosteroids (BRs) are involved in regulating the leaf inclination. IAA is critical to the development of leaf morphology in maize, and interacts with light (white, red, and blue) or ethylene in regulation of leaf declination (Fellner et al., 2003). In addition, the regulatory pathways of auxin signaling and BR biosynthesis are also included (Liu et al., 2019), and GAs genes interact with BR genes to modulate the leaf angle (Li et al., 2020). Two rice (*Oryza sativa* L.) auxin response factors (ARFs), OsARF6 and OsARF17, which are highly expressed in lamina joint tissues, control flag leaf angle in response to auxin (Huang et al., 2021). And rice LEAF INCLUSION1 (LC1), an IAA amide synthase, maintains auxin homeostasis by binding excess IAA with various amino acids, and then regulates the leaf angle (Zhao et al., 2013). In addition, Li et al. found that ABA regulate lamina joint inclination in rice by the BR biosynthesis pathway and BR signal transduction (Li et al., 2019a).

Basic helix-loop-helix (bHLH) transcription factor (TF) is a major regulatory factor, which is one of the largest TF family, and widely distributed among eukaryotic kingdoms. The bHLH domain generally contains approximately 60 amino acids and possesses two functionally distinct regions: a basic region and a helix-loop-helix (HLH) region (Guo and Wang, 2017). The basic region is located at the N-terminus along with a DNA-binding motif, and the HLH region contains two amphipathic α-helices separated by a loop region of variable length which could act as a dimerization domain that promotes protein-protein interactions and forms homo-dimers or hetero-dimers (Massari and Murre, 2000).

Studies have clarified that bHLH TFs play a regulatory role in plant growth and development (Heang and Sassa, 2012; Endo et al., 2016), response to a variety of abiotic stresses (Li et al., 2007; Ogo et al., 2007) and signal transduction (Friedrichsen et al., 2002; Huq and Quail, 2002; Zhang et al., 2011). The rice bHLH TF OsBIM1 has been reported to positively regulate leaf angle by promoting brassinolide signal transduction (Tian et al., 2021). The maize bHLH (ZmbHLH) TF PTF1 can promote lateral root elongation and assist with abscisic acid (ABA) biosynthesis, signal transduction, and drought tolerance (Li et al., 2019b). In addition, *bHLH* genes in plants have also found to be involved in the biosynthesis pathways of anthocyanins (Wang et al., 2018).

At present, the regulation functions of bHLH TF of leaf angle are widely studied in rice, but important questions remain. In this study, we obtained the *ZmbHLH112* mutant *bhlh112* by ethyl methane sulfonate (EMS) mutagenesis of wild-type B73, which displays a relatively small leaf angle. Maize mutant *bhlh112* and wild-type B73 were analyzed using RNA-Seq sequencing. Our findings will provide useful information for further studies in maize plant type breeding to clarify the functions of bHLH TF in diverse growth and development processes.

## 2 Materials and methods

### 2.1 Plant materials and planting conditions

Mutant *bhlh112* (EMS3-03e012) with altered expression of maize leaf angle was obtained from the maize EMS mutant library of Qilu Normal University (http://elabcaas.cn/memd/). The mutant *bhlh112* was planted in the experimental field of Gansu Agricultural University, we used mutant *bhlh112* to continuously backcross B73 for 2 generations and then self-bring for one generation to clear the mutant background. We then used the Sanger Method to preserve the mutated sites during background removal and observe the mutant phenotypes. The experiment was arranged in a complete randomized block design with replicated three time, and technique of the alternately planting of wide (70 cm) and narrow (40 cm) row spacing was used with plant spacing of 25 cm (75000 seeds ha^-1^). B73 germplasm material was provided by the Maize Breeding Research Group of the Agricultural College of Gansu Agricultural University. The experiment was carried out at the State Key Laboratory of Aridland Crop Science, Gansu Agricultural University.

### 2.2 Bioinformatics analysis of *ZmbHLH112*

We used the NCBI database to find the gene sequence and amino acid sequence encoded by *ZmbHLH112*, DNAMAN software to analyze the *ZmbHLH112* sequence and sequence alignment, ClustalW programs to perform multiple alignment of amino acid sequences, and MEGA7.0 software to construct multiple alignment results generated by the ClustalW Phylogenetic tree; Using the Neighbor-joining method with select Pairwise deletion, bootstrap set to 1000.

### 2.3 Mutant phenotype analysis

After pollination, three plants of wild-types and mutant with look similar were selected in each plot, respectively, the field phenotypic data (plant height, ear height, chlorophyll, stem diameter, and branch number of tassels) were measured; Meanwhile, ten wild-type and mutants with similar appearance were selected and their ear leaf angle, a leaf angle above the ear, a leaf angle below the ear were measured respectively. Mature ears were harvested at the end of September of the same year and dried to safe moisture before threshing. At that time, the single ear weight, ear length, ear diameter, grain type, grain color, and 100 grain weight were measured, the values shown are averages from three biological replicates. Leaf area size (LAS= LL x LW x 0.75) and leaf direction value (LOV = ∑ (90 - θ i) x (Lf/LL)/N were calculated; θ i: leaf angle value; Lf: leaf spacing; LL: leaf length; N: number of leaves above the ear) (Zhang et al., 2021a).

### 2.4 Identification of the homozygous mutant *bhlh112*

We used mutant *bhlh112* to continuously backcross B73 for 2 generations and then self-bring for one generation as the experimental material, DNA was extracted from V3 stage leaves of the mutant *bhlh112* and wild-type B73. Primer premier 5.0 was used to design primers: forward primer: GGAGTTCTGGCACCCTACAT; reverse primer: TTTGGGCGGCATCATGATTT. Using wild-type and mutant DNA as the template, PCR amplification was carried out with Easy-Taq enzyme. The reaction system (20 μL) included Easy Taq 10 μL, ddH_2_O 7 μL, forward and reverse primers 1 μL each, and DNA template 1 μL. Amplification conditions were pre-denaturation at 94°C for 4 min, 94°C for 1 min, 56°C for 30 s, 72°C for 1 min, after 35 cycles, extension at 72°C for 10 min. PCR amplification products were sent to Shanghai Sangon Biotech Company for sequencing, and the mutation sites were determined by DNAMAN analysis.

### 2.5 Expression pattern analysis of *ZmbHLH112*

#### 2.5.1 Extraction of total RNA and synthesis of first strand cDNA

The root, stem, old leaves, new leaves, and the leaf occipital part of the second leaf of maize B73 at the V3 stage were collected and frozen in liquid nitrogen. Three biological replicates for each sampling part in this study.The SteadyPure plant RNA extraction kit was used to extract total RNA and the RNA concentration, purity, and integrity were examined using advanced molecular biology equipment. cDNA (including gDNA) was synthesized by M-MLV (Moloney murine leukemia virus) reverse transcription kit, provided by Accurate Biology Company. Reverse transcription conditions: 37°C for 15 min, 85°C for 5 sec, and 4°C.

#### 2.5.2 Quantitative real-time PCR (qRT-PCR)

We used the SYBR ^®^ Green Pro Taq HS Premixed qPCR kit was used for qRT-PCR, and the internal reference gene Actin (forward primer: TGAAACCTTCGAATGCCCAG, reverse primer: GATTGGAACCGTGTGGCTCA; *ZmbHLH112*: forward primer: TAACGGCGACACGAAGCAAGAC, reverse primer: CTCCAAATGTAGGGTGCCAGAACTC). The reaction system was 20 µL: 2xSYBR ^®^ Green Pro Taq HS Premix 10 µL; cDNA 1 µL, forward primer 0.4 µL and reverse primer 0.4 µL, RNase free water 8.2 µL. This was amplified by Illumina qRT-PCR; 2^-ΔΔCT^ calculated the relative expression of genes (Livak and Schmittgen, 2001). Reaction conditions: 95 °C for 30 sec, followed by cycling for 40 rounds of 95°C for 5 sec, 60°C for 30 sec.

### 2.6 Screening the optimal concentration of exogenous hormones

The maize seeds were planted with vermiculite in a flower pot with a diameter of 10 cm and placed in an artificial climate room (12 h light/12 h dark, 28°C, 65% RH) to determine the phenotypic and physiological characteristics of the seedlings. Two hormone treatment groups were set up in this experiment; (1) ABA application at four concentrations: B73 and *bhlh112* with 0 μmol·L^-1^ (control), 1 μmol·L^-1^, 10 μmol·L^-1^ and 50 μmol·L^-1^; (2) IAA application at five concentrations: B73 and *bhlh112* are set to 0 μmol·L^-1^ (control), 1 μmol·L^-1^, 10 μmol·L^-1^, 100 μmol·L^-1^ and 500 μmol·L^-1^. Treatment with exogenous plant hormones was carried out from the normal watering to the V1 stage of the seedlings. The treatment was conducted once every other day, with 50 mL poured each time, and the leaves of the corn seedlings sprayed with the corresponding hormones. The leaf angle of each treatment was measured at the V3 stage to screen the optimal concentration of exogenous hormones. 2 cm of the leaf occipital part of the V3 stage at the optimal concentration was collected and frozen in liquid nitrogen, and sent it to Lianchuan biological company for sequencing.

### 2.7 RNA-seq library construction and sequencing

For RNA-seq analysis, the second leaf occipital part of the wild-type and mutant plants at the V3 stage were harvested and frozen in liquid nitrogen. Three biological replicates for each genotype and three pooled samples for each replicate were tested in this study. The total RNA of the sample was extracted with TRIzol (Thermo Fisher, 15596018), and the RNA was isolated and purified according to the manufacturer’s instructions. The quantity and purity of total RNA was controlled with NanoDrop ND-1000 (NanoDrop, Wilmington, DE, USA), and the integrity of RNA was detected with Bioanalyzer 2100 (Agilent, CA, USA); Illumina Novaseq™ 6000 (LC Bio Technology CO., Ltd. Hangzhou, China) was used for double ended sequencing according to the standard operating procedures. Clean Data bases produced by each sample more than 43 million; About 94.37% - 97.95% of the clean data can be compared to the number of reads on the reference genome (RefGen_v4) of B73, about 90.48% - 94.27% can only be compared to the number of reads at one position of the genome, and only about 3.51% - 5.14% of the clean data can be compared to the number of reads at multiple positions of the genome. The size of the filtered data accounted for 97.69% of the original data size. The percentage of bases with a quality value of Q≥30 is 97.74% and above. The data presented in the study are deposited in the SRA at NCBI repository, accession number PRJNA856349.

### 2.8 qRT-PCR validation of the DEGs

In order to verify the reliability of the DEGs, the differentially expressed genes in different comparison groups were selected for qRT-PCR. The method was the same as in 2.5.2; the primer sequence in the **Table 1**.

**Table 1:** Primer design sequence.

Gene name	Forward Primer (5’→3’)	Reverse Primer (5’→3’)
Zm00001d009985	GCAAGGTGCGCTCCTTCTTCTG	ACGAGAGAGACGAGGACGACAAG
Zm00001d022435	CGATGACGACTACGACTACGACTTG	TTGATCCTGATGGCTCCCGAGTC
Zm00001d016719	CACCAAGCTGCTGCTGCTCATG	GCTCTTGCCGTTGACCCTGAAG
Zm00001d018150	AAGAAGGTTGGTAGCGGCAAGAAG	CGACCGGATTAGCTTCCACCATG
Zm00001d019629	GCGAGATGAGGCTCAGCAACTTC	CGCAACGCTTCCAGGTACTCAG
Zm00001d009687	ACCAATCTCCACCGCCTCAT	GAAGGCATGGACGAGGACGA
Zm00001d032024	CGGGAACAAGTGGTCGCTCATC	ACGTGCGTGTTCCAGTAGTTCTTG
Zm00001d007175	TGGACGAGCCACTGATCTCC	CGGTGGACGAGGAGGTTGAT

### 2.9 Co-expression network analysis for module construction

Co-expression networks were constructed using the WGCNA package in R. We selected genes with FPKM ≥ 10, gene expression values were imported into WGCNA to construct co-expression modules with default settings, TOM Type was unsigned, used the picksoftthreshold function to select the appropriate soft threshold β = 18, selected the threshold value close to 0.9 of the fitting curve, and constructed the scale-free network distribution. We associated the merged modules with phenotypic traits, selected the genes of the core module top30 according to the weighted values, constructed the protein interaction network between genes with the string website, and drew the network diagram with Cytoscape function.

## 3 Results

### 3.1 Identification of the *ZmbHLH112* Gene

**Figure 1:**
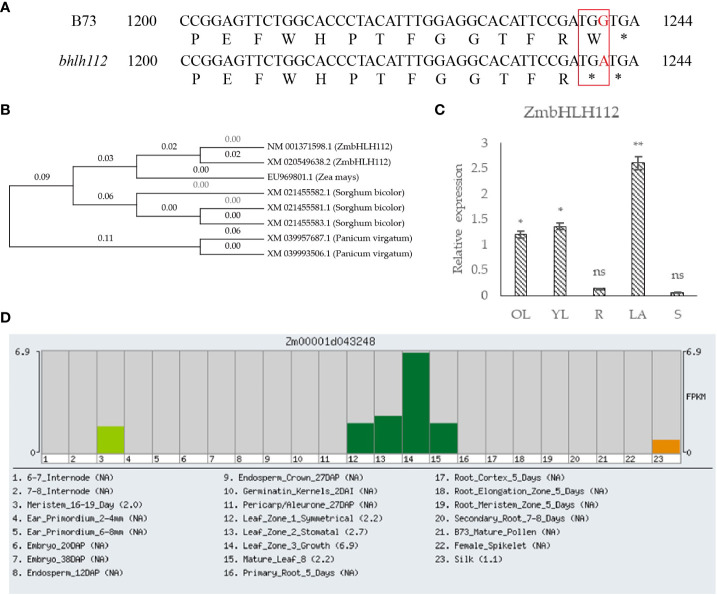
Analysis of the *ZmbHLH112* gene in maize. **(A)** Partial sequence alignment of the *ZmbHLH112* gene in mutants *bhlh112* and B73. **(B)** Evolutionary tree of the *ZmbHLH112* gene. **(C)** Relative expression of *ZmbHLH112* in different tissues. **(D)** Gene Expression Atlas of the *ZmbHLH112* gene from Maize GDB. OL, old leaves; YL, young leaves; R, roots; LA, the leaf occipital part of the second leaf at the V3 stage; S: stems. ** indicates P < 0.01, * indicates P < 0.05. ns indicates not significant.

The *ZmbHLH112* (*Zm00001d043248*) gene (LOC100277655) is located on maize chromosome 3, with a total length of 1525bp, including 1158bp CDs sequence, encoding 385 amino acids. The protein sequence of *ZmbHLH112* was analyzed by Blast in the NCBI database; we found that it had the highest homology with sorghum bicolor (XM_021455582.1 and XM_021455581.1). The above plant protein sequences were compared by MEGA7.0 software, and then a phylogenetic tree was constructed (**Figure 1B**). The 1241th base G of *ZmbHLH112* gene in the mutant *bhlh112* was replaced with A (**Figure 1A**), whereby tryptophan (TGG) was mutated into termination codon (TGA). The mutation at this base leads to the differences in maize phenotype and physiological structure. In order to verify the expression specificity of the *ZmbHLH112* gene in different maize tissues, the expression patterns of the *ZmbHLH112* gene in maize roots, stems, old leaves (second leaf at the V3 stage), young leaves (fourth leaf at the V3 stage) and the leaf occipital part of the second leaf were detected by quantitative real-time polymerase chain reaction (qRT-PCR) analysis. *ZmbHLH112* had the highest relative expression of the leaf occipital part of the second leaf; relative expression was the lowest in roots and stems (**Figure 1C**). The *ZmbHLH112* gene was related to the development of meristem, leaf, and silk according to the Maize GDB website (https://www.maizegdb.org/) (**Figure 1D**). These results showed that this gene could regulate the development of maize leaves and the leaf occipital part.

### 3.2 Phenotypic and grain characters of *bhlh112*

**Figure 2:**
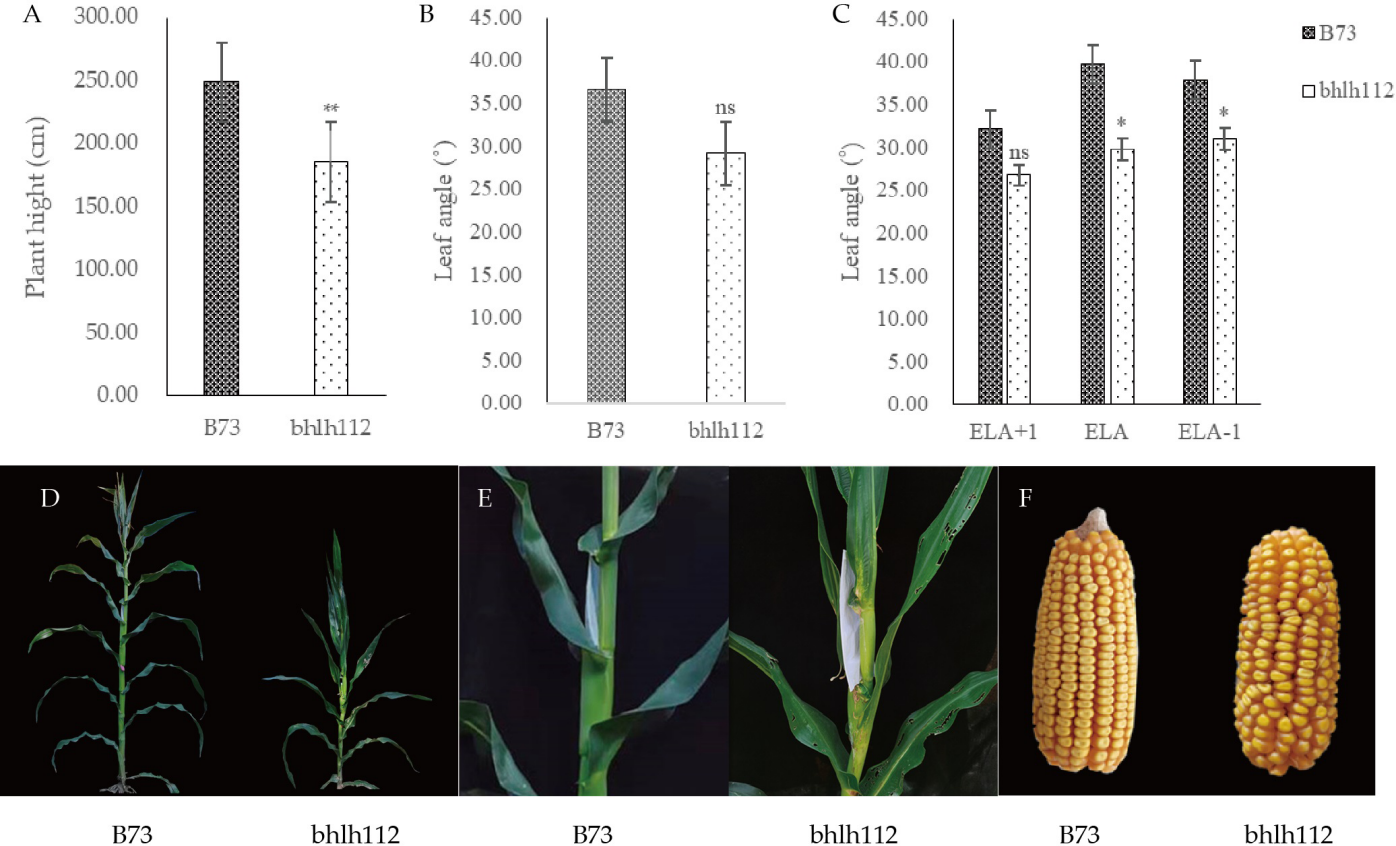
Analysis of index of wild-type and mutant *bhlh112*. **(A)** Plant height of mutant and wild-type; **(B)** Leaf angle of mutant and wild-type; **(C)** Three ear-leaf-angle of mutant and wild-type at the mature stage. ELA: ear leaf angle; ELA+1: a leaf angle above the ear; ELA-1: a leaf angle below the ear; ns indicates not significant; **(D)** Plants at silking stage; **(E)** Corresponding leaf angle in **(D)**; **(F)** Mature ear of wild type and mutant. * indicates P < 0.05. ** indicates P < 0.01.

Phenotypic analysis of mutant *bhlh112* and its wild-type B73 showed that significant reductions in plant height and ear height were observed in the *bhlh112* mutant when compared to the wild-type, with 31.93% and 19.41% reductions observed at the mature stage, respectively (**Figures 2A, D**). The leaf angles also significantly differed; the average leaf angle of the mutant was smaller than that of the wild-type (**Figure 2B**). We measured the ear leaf angle, a leaf angle above the ear, and a leaf angle below the ear, and observed 16.70%, 25.00%, and 18.36% reductions in these leaf angles, respectively, in *bhlh112* at the mature stage (**Figures 2C, E**). Compared to the wild-type, the single ear weight, ear length, and ear diameter of the mutant decreased by 35.96%, 22.09%, and 6.48%, ear rows, number of rows and ear shaft weight decreased 10.28%, 6.96% and 22.89%, respectively; And, the 100-kernel weight increased by 3.25% (**Figure 2F**). In summary, the phenotypes of mutants and wild-type differed in many dimensions (**Tables 2**, **3**).

**Table 2:** Field phenotypic traits of mutant *bhlh112* and B73.

Traits	B73	*bhlh112*
Plant height/cm	250.39 ± 0.87a	170.44 ± 4.94b
Ear height/cm	108.78 ± 1.95a	87.67 ± 1.26b
Stem diameter/mm	22.29 ± 0.38a	18.03 ± 0.69b
Tassel branch number	8.89 ± 0.35a	3.44 ± 0.44b
Chlorophyll	50.14 ± 1.64a	34.92 ± 0.69b
Leaf length/cm	78.54 ± 2.08a	63.34 ± 1.61b
Leaf width/cm	9.23 ± 0.18a	7.16 ± 0.23b
Leaf orientation value	5.35 ± 0.28a	7.03 ± 0.43b
Leaf area size/cm2	545.56 ± 23.84a	341.78 ± 17.31b

a, b indicated significant differences between materials. P < 0.05.

**Table 3:** Field grain traits of mutant *bhlh112* and B73.

Traits	B73	*bhlh112*
Ear length/cm	10.87 ± 0.49a	8.47 ± 0.70b
Ear coarse/cm	3.97 ± 0.32a	3.37 ± 0.25b
Ear rows	16.67 ± 1.15a	12.67 ± 1.15b
Number of rows	26.67 ± 1.15a	22.33 ± 1.53a
Ear weight/g	81.52 ± 6.98a	52.21 ± 1.06b
Kernel weight/g	66.66 ± 3.27a	45.28 ± 0.59a
100-kernel weight/g	41.53 ± 3.56a	44.68 ± 0.61a
Shaft weight/g	14.86 ± 1.80a	6.92 ± 0.09b
Shaft coarse/cm	2.77 ± 0.21a	2.27 ± 0.21b

a, b indicated significant differences between materials. P < 0.05.

### 3.3 Screening of optimum concentrations of different exogenous plant hormones

**Figure 3:**
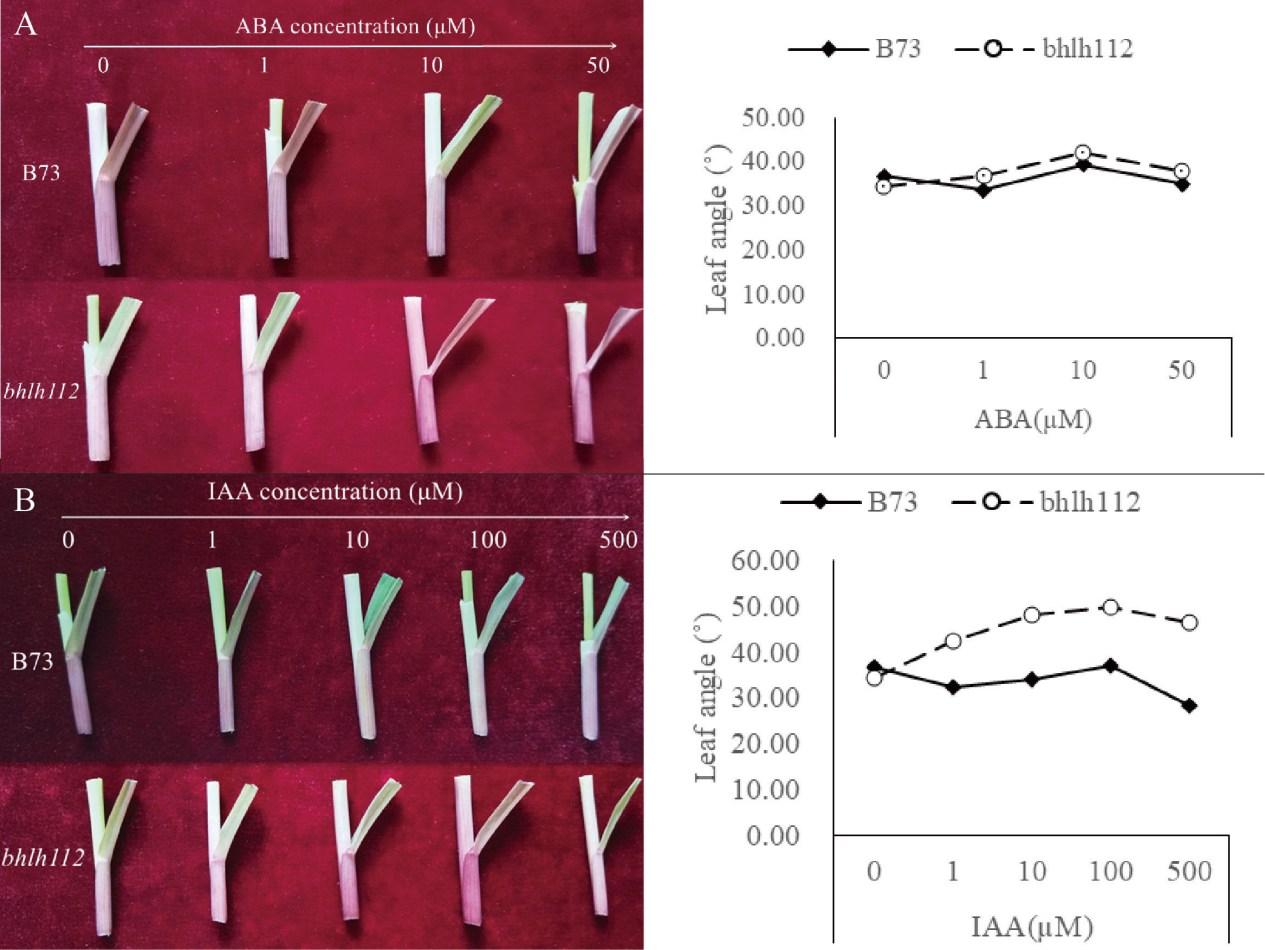
Effects of ABA and IAA on leaf angle in maize. **(A)** Changes in leaf angle from different concentrations of ABA in mutant and wild-type V3 stage. **(B)** Changes in leaf angle from different concentrations of IAA in the mutant and wild-type V3 stage.

In order to further understand the regulation of bHLH TF of maize leaf angle and the effect of exogenous hormones on leaf angle development, we treated mutant *bhlh112* and the wild-type with the exogenous hormones ABA and IAA, and observed leaf angles at the V3 stage. When treated with 0 µmol·L^-1^ ABA (control), the leaf angle of B73 was larger than that of *bhlh112* (**Figure 3A**), which was consistent with the results from field data, which also showed a smaller leaf angle of the mutant *bhlh112*. However, with increasing ABA concentration, the leaf angle of *bhlh112* and B73 increased first and then decreased, and reached a maximum at 10 µmol·L^-1^, indicating that this is the preferred treatment concentration. At this concentration, the leaf angles were 42.15° and 39.33° in the mutant *bhlh112* and the wild-type, respectively, which represents a respective increase of 22.29% and 7.325%, compared with the control. When treated with increased concentrations of exogenous IAA, the leaf angle of *bhlh112* and B73 increased first and then decreased (**Figure 3B**). When exogenous IAA was 100 µmol·L^-1^, the leaf angles of *bhlh112* and B73 reached the maximum, 49.92° and 37.03° respectively, which was an increase of 44.83% and 1.05% respectively compared with the control. In general, the mutant *bhlh112* was more sensitive to different exogenous plant hormones than was the wild-type B73.

### 3.4 Transcriptomic analyses of *bhlh112* and B73

**Figure 4:**
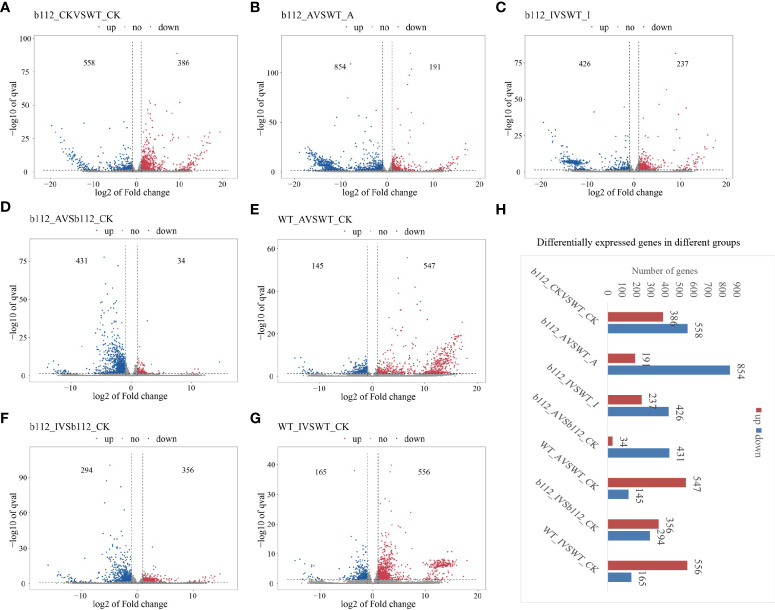
DEGs between *bhlh112* and the wild-type. **(A)** Number of DEGs of *bhlh112* and B73 under the control treatment; **(B)** Number of DEGs of *bhlh112* and B73 under 10 µmol·L^-1^ exogenous ABA treatment; **(C)** Number of DEGs of *bhlh112* and B73 under 100 µmol·L^-1^ exogenous IAA treatment; **(D)** Number of DEGs of *bhlh112* under 0 µmol·L^-1^ and 10 µmol·L^-1^ exogenous ABA treatment; **(E)** Number of DEGs of B73 under 0 µmol·L^-1^ and 10 µmol·L^-1^ exogenous ABA treatment; **(F)** Number of DEGs of *bhlh112* under 0 µmol·L^-1^ and 100 µmol·L^-1^ exogenous IAA treatment; **(G)** Number of DEGs of B73 under 0 µmol·L^-1^ and 100 µmol·L^-1^ exogenous IAA treatment; **(H)** DEGs across different groups. control = CK = 0 µmol·L^-1^ ABA = 0 µmol·L^-1^ IAA.

In order to understand the transcriptome network underlying the *ZmbHLH112* gene mutation, high-throughput RNA-seq was performed for mutant *bhlh112* and the wild-type B73. Fold changes > = 2 (|log2FC | > = 1) was used as the change threshold, and q < 0.05 (q value is the correction value of p value) was used as the standard for screening DEGs. In the control group, a total of 944 DEGs were identified. Among them, 386 genes were significantly up-regulated, and 558 genes were significantly down-regulated, accounting for 40.89% and 59.11%, respectively, of the DEGs (**Figure 4A** and **Table S1**). In *bhlh112* and B73 under 10 µmol·L^-1^ exogenous ABA treatment, a total of 1045 DEGs were identified, with 191 genes up-regulated and 854 genes down-regulated, accounting for 18.28% and 81.72% of the total DEGs, respectively (**Figure 4B** and **Table S2**). When *bhlh112* and B73 were treated with 100 µmol·L^-1^ exogenous IAA, a total of 663 DEGs were identified, and the number of up-regulated and down-regulated genes were 237 and 426, accounting for 35.75% and 64.25% of the total DEGs, respectively (**Figure 4C** and **Table S3**); *bhlh112* and B73 were treated with 0 µmol·L^-1^ and 10 µmol·L^-1^ exogenous ABA, and a total of 465 and 692 DEGs were identified, of which the numbers of up-regulated genes were 34 and 431, accounting for 7.31% and 92.68% of the total DEGs, respectively, and the numbers of down-regulated genes were 547 and 145, accounting for 79.05% and 20.95% of the total DEGs respectively (**Figures 4D, E**, **Tables S4**, **S5**). *bhlh112* and B73 were treated with 0 µmol·L^-1^ and 100 µmol·L^-1^ exogenous IAA, and a total of 650 and 721 DEGs were identified respectively, of which the numbers of up-regulated genes were 356 and 556, accounting for 54.77% and 45.23% of the total DEGs, respectively, and the numbers of down-regulated genes were 294 and 165, accounting for 77.12% and 22.88% of the total DEGs, respectively (**Figures 4F, G**, **Tables S6**, **S7**). Further observation of the number of DEGs between each comparison group showed that there were some differences in the responses of the mutant *bhlh112* and B73 to different exogenous plant hormones (**Figure 4H**).

### 3.5 GO terms and KEGG pathways analysis of leaf angle in *bhlh112*

**Figure 5:**
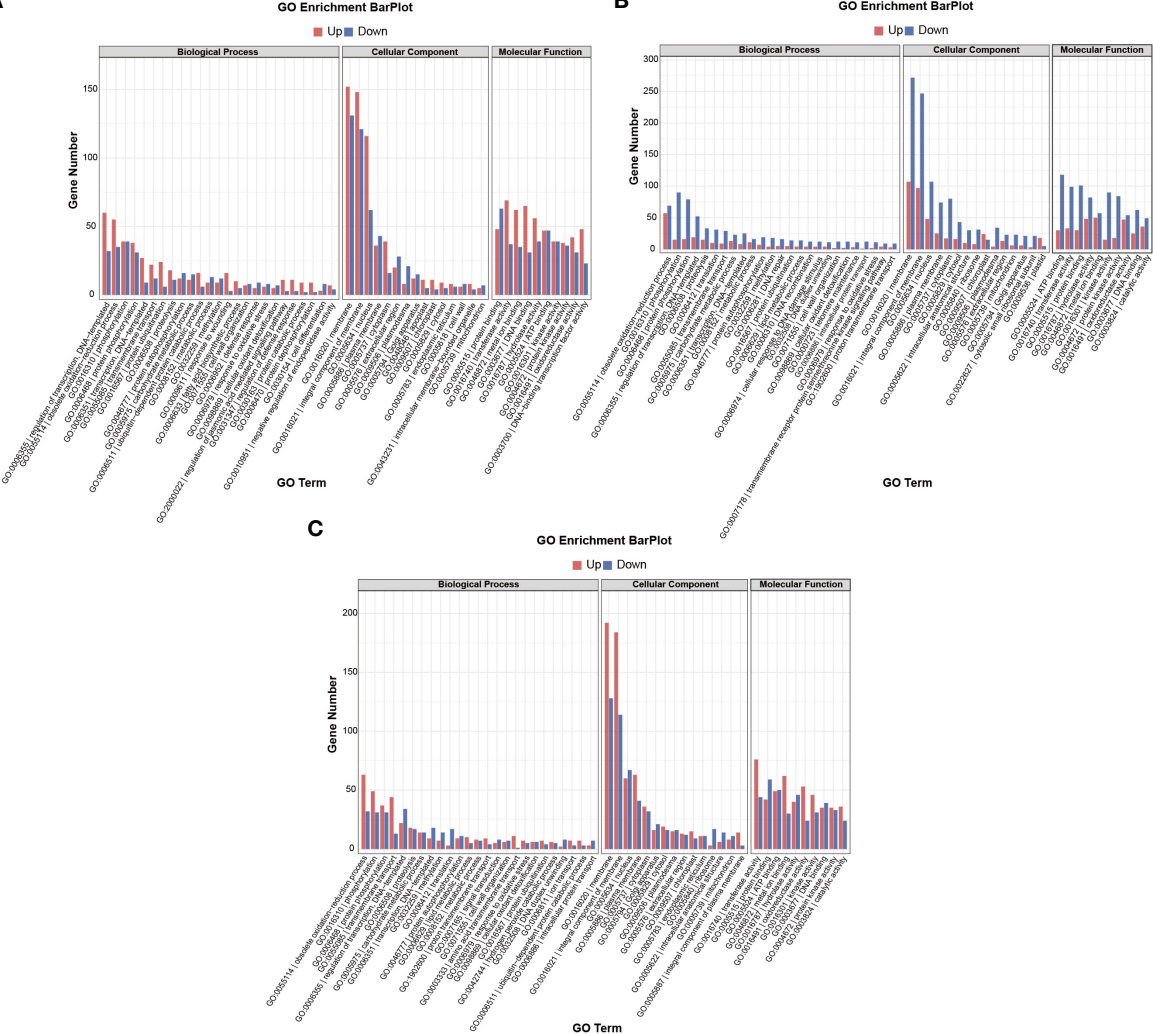
GO term annotations of DEGs in B73 and *bhlh112* under the different treatments. **(A)** b112_CKvsB73_CK; **(B)** b112_AvsB73_A; **(C)** b112_IvsB71_I. CK = control, A = exogenous ABA, I = exogenous IAA.

**Figure 6:**
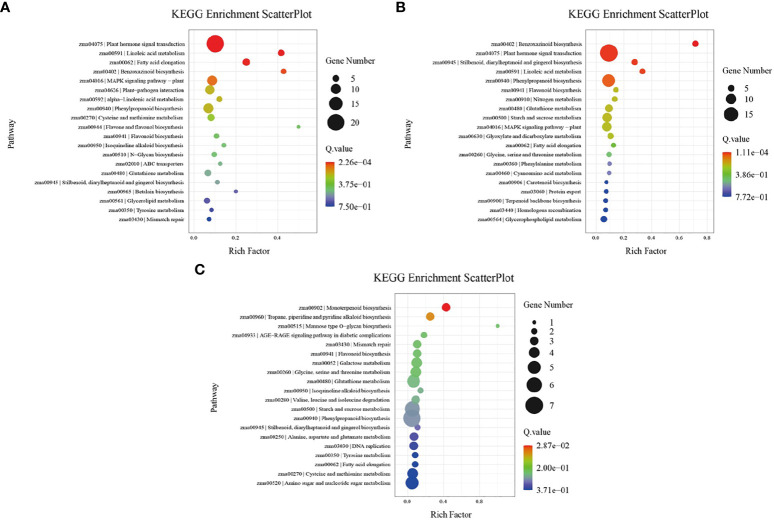
KEGG pathway enrichment analysis of DEGs in B73 and *bhlh112* under the different treatments. **(A)** b112_CKvsB73_CK; **(B)** b112_AvsB73_A; **(C)** b112_IvsB71_I. CK = control, A = exogenous ABA, I = exogenous IAA.

Both GO terms and KEGG pathways were used to elucidate the functional annotations of the DEGs (**Figures 5**, **6**). Annotation functions of GO terms were mainly divided into three categories: biological processes (BP), cellular components (CC), and molecular functions (MF). The up-regulated DEGs were significantly represented in 209 BP terms, 114 CC terms, and 31 MF terms; the down-regulated DEGs were significantly represented in 33 BP terms, 312 CC terms, and 206 MF terms. Compared with the wild-type B73, in the mutant *bhlh112*, the most significantly enriched GO terms mainly included the BP terms “oxylipin biosynthetic process”, “aromatic compound biosynthetic process”, “regulation of jasmonic acid mediated signaling pathway” and “regulation of defense response”; the CC terms “extracellular region” and “apoplast”; the MF terms “dioxygenase activity”, “oxidoreductase activity”, “O-methyltransferase activity” and “serine-type endopeptidase inhibitor activity” (**Figure 5**). KEGG enrichment analysis revealed that “Plant hormone signal transduction”, “MAPK signaling pathway-plant” and “Plant-pathogen interaction” were the most overrepresented pathways in the up-regulated DEGs, while “DNA replication”, “Lipid metabolism”, “Nucleotide excision repair”, and “Mismatch repair” were the main pathways in the down-regulated DEGs (**Figure 6**). These results showed that the expression level of genes related to plant hormone signal transduction, MAPK signaling pathway-plant, and response to wounding were increased in the mutant *bhlh112*, and the expression level of genes related to genetic information processing were inhibited.

### 3.6 Pathway enrichment analysis of *bhlh112* and B73 with exogenous ABA

We performed pathway enrichment analysis on all DEGs in wild-type B73 and the mutant *bhlh112* (**Figures 5B**, **6B**). Under ABA treatment, compared with B73, *bhlh112* had 61, 3, and 123 significantly up-regulated DEGs involved in biological processes, cell composition, and molecular functions (q<0.05), and 76, 21, and 207 significantly down-regulated DEGs for these three categories. For BP, DEGs were enriched in the “obsolete oxidation-reduction process”, “tryptophan biosynthetic process”, and “negative regulation of endopeptidase activity”. The enriched CC terms were primarily “cytosolic small ribosomal subunit”, “anthranilate synthase complex”, and “plasmodesma”. The enriched MF terms were “oxidoreductase activity”, “hydrolase activity”, “protein kinase activity”, “phosphate ion transmembrane transporter activity”, and “catalytic activity”. Further KEGG enrichment analysis revealed that up-regulated genes were enriched in “Stilbenoid, diarylheptanoid and gingerol biosynthesis”, “Linoleic acid metabolism”, and “Phenylpropanoid biosynthesis”; down-regulated genes were enriched in “Benzoxazinoid biosynthesis” and “Plant hormone signal transduction”. These results suggest that exogenous ABA may be involved in leaf angle development of the mutant *bhlh112* through plant hormone signal transduction, lipid metabolism, and biosynthesis of secondary metabolites.

### 3.7 Pathway enrichment analysis of *bhlh112* and B73 with exogenous IAA

Under IAA treatment (**Figures 5C**, **6C**), compared with B73, the mutant *bhlh112* had the significantly enriched BP terms “amino acid transmembrane transport”, “obsolete oxidation-reduction process”, “cellulose biosynthetic process”, “exocyst assembly”, “cell surface receptor signaling pathway” and “hexose transmembrane transport”; enriched CC terms were “plasma membrane” and “plasmodesma”; MF terms were “oxidoreductase activity”, “monooxygenase activity”, “amino acid transmembrane transporter activity”, “carbohydrate:proton symporter activity” and “protein histidine kinase binding”.

### 3.8 Validation of DEGs by qRT-PCR analysis

**Figure 7:**
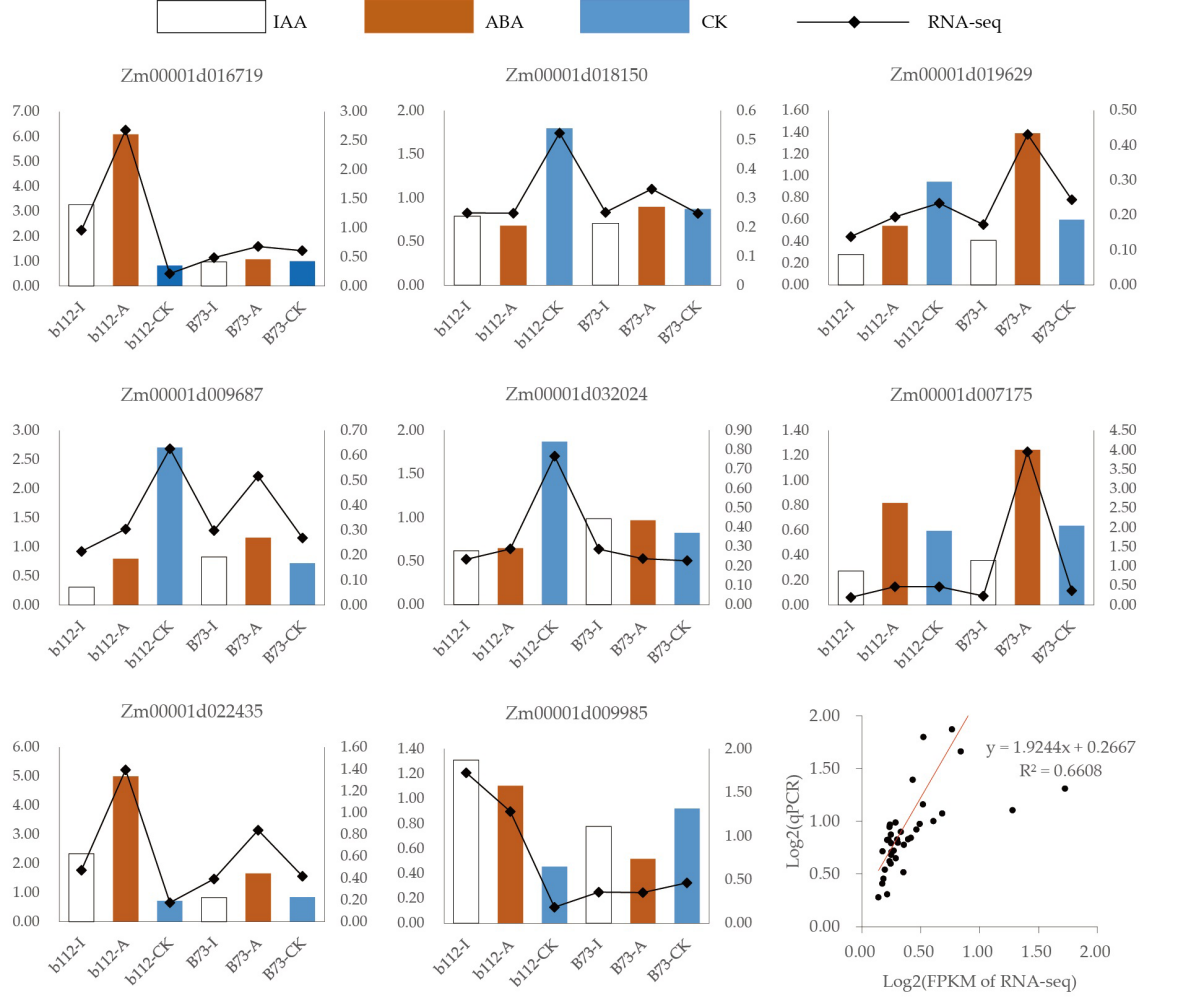
qRT-PCR was performed using 8 randomly selected DEGs. Bar and line graphs represent the qRT-PCR and RNA-Seq data, respectively.

The expression levels of some DEGs were evaluated by qRT-PCR in order to validate the reliability of the RNA-seq data (**Figure 7**). We found that 8 DEGs exhibited the same trends in both the RNA-Seq and qRT-PCR results, indicating that the RNA-seq data was reliable (y = 1.9244x+0.2667, R^2 = 0.6608).

### 3.9 Weighted correlation network analysis (WGCNA)

#### 3.9.1 Construction of modules associated with leaf angle of maize based on WGCNA

**Figure 8:**
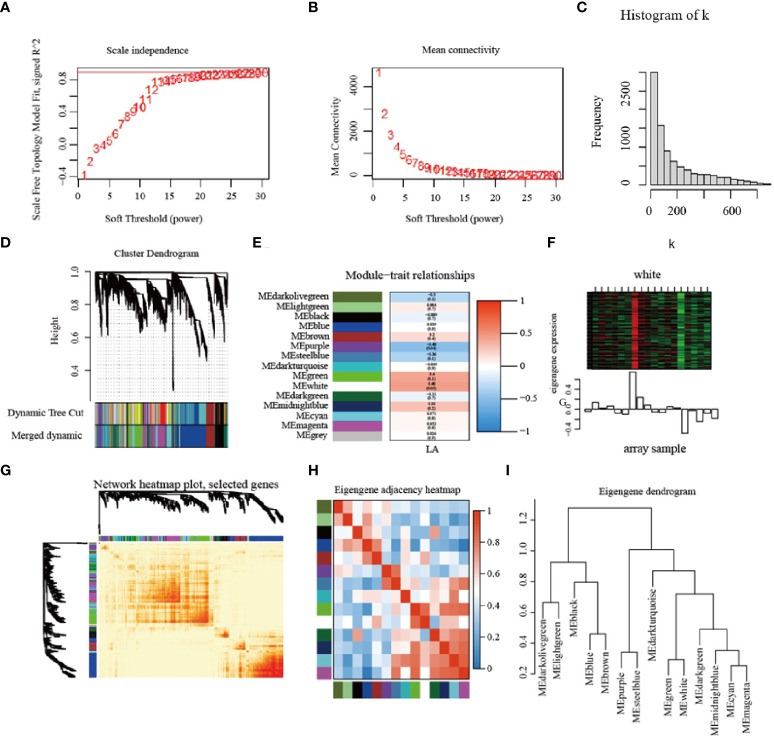
Construction of modules based on weighted gene co-expression network analysis. **(A–C)** Soft power value screening of differentially expression genes for LA related co-expression modules; **(D)** Establishment of the cluster dendrogram in LA co-expression mod-ules. **(E)** The correlation between modules and LA; **(F)** White module; **(G)** Network heatmap; **(H, I)** Module clustering. LA = leaf angle.

In order to identify the correlation between the genes obtained by RNA-seq and the phenotypic data of maize leaf angle (LA), the DEGs with FPKM ≥ 10 were selected, and a total of 8994 DEGs were screened to construct the co-expression network. Used the threshold value close to 0.9 of the fitting curve (**Figures 8A, B**), and constructed the scale-free network distribution (**Figures 8C, D**). A total of 15 modules meeting the conditions were screened, of which the white module had the highest correlation with the leaf angle of maize (**Figures 8E, F**). These 15 modules were analyzed by correlation and cluster analysis (**Figures 8G-I**), and they were clustered into 3 categories at 1.0

#### 3.9.2 Identification of hub genes and construction of the PPI network

**Figure 9:**
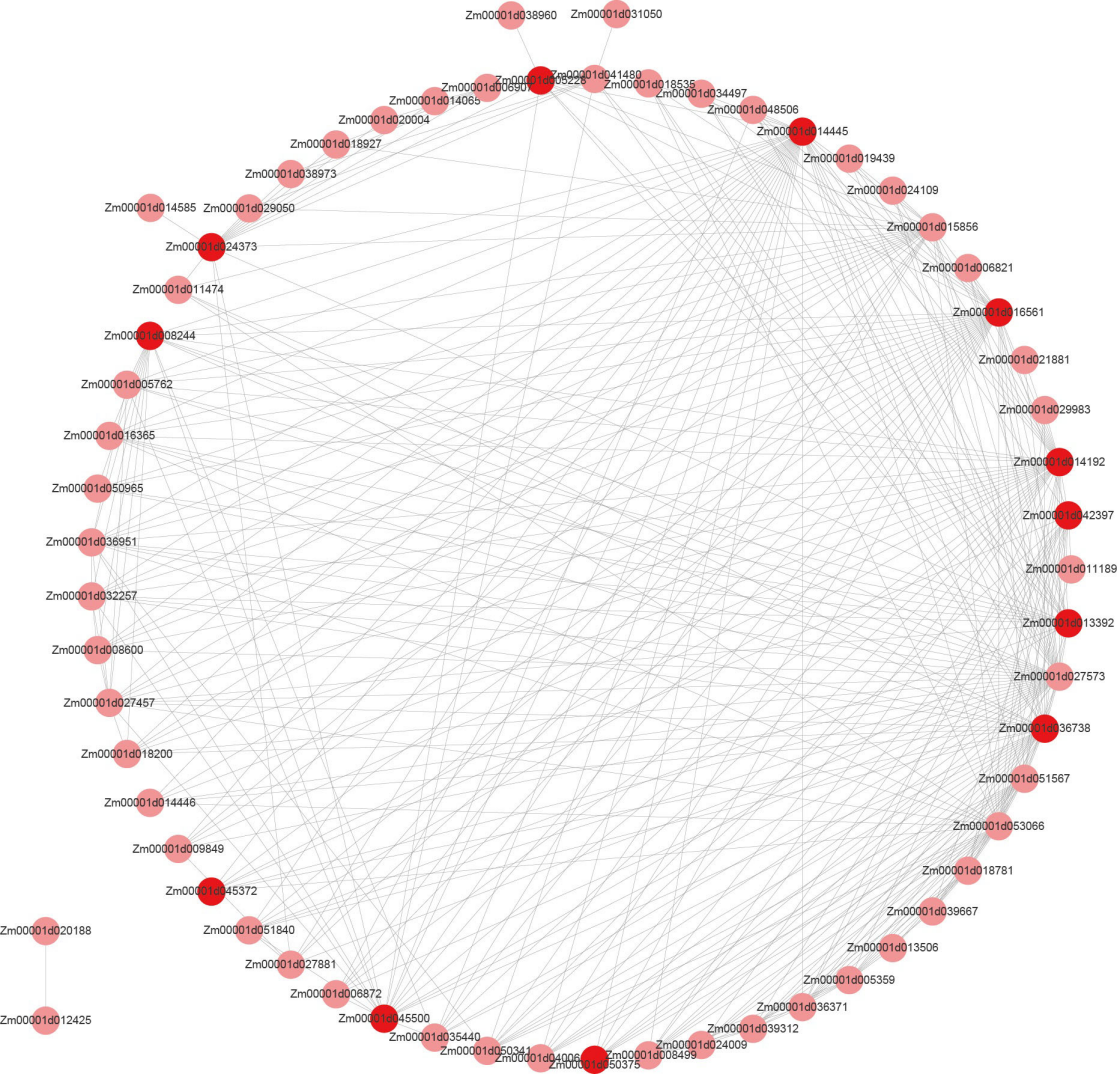
Co-expression regulatory network analysis of the white module. Red represents hub genes.

**Figure 10:**
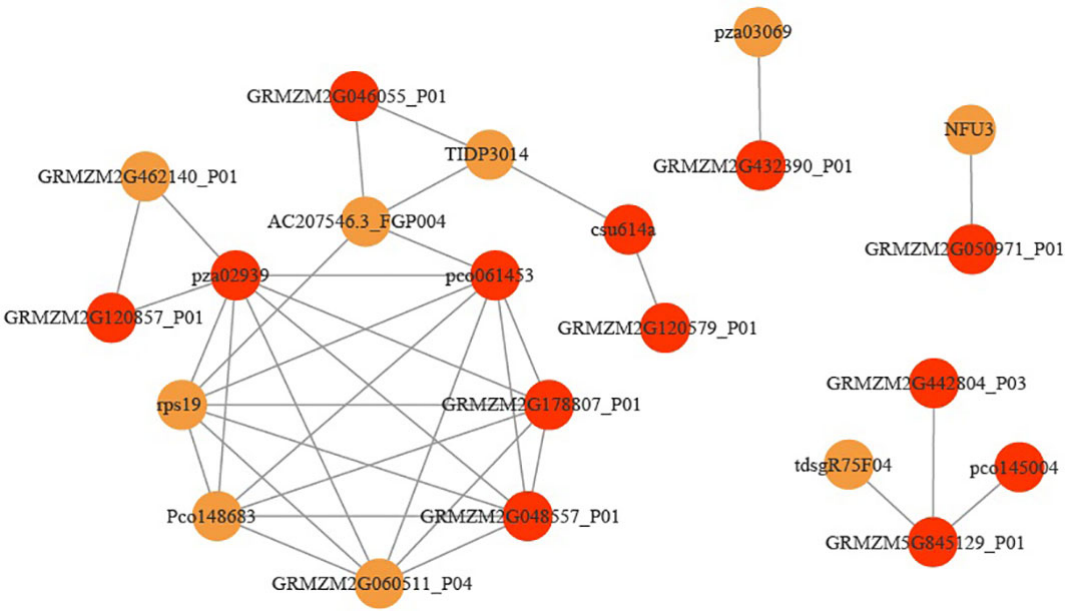
Co-expression regulatory network analysis of the hub genes. Red represents hub genes.

In order to further screen the genes related to leaf angle, a total of 63 genes were screened for the white module based on | GS | >0.2 and | MM | >0.8 (GS: Gene significance for body weight; MM: Module Membership in white module) (**Table S8**). A protein-protein interaction (PPI) network was constructed using the STRING database with a combined score of >0.4. In addition, the PPI network was constructed with the genes with the weighted value of top30 (**Figure 9**). The PPI relationship between them was predicted by using the STRING database, and retaining the combined_score > 0.4, the PPI network of 12 hub genes was obtained (**Table 4**). The interconnected lines between genes represent co-expression interrelations, and the PPI network diagram was drawn by Cytoscape (**Figure 10**).

**Table 4:** The 12 hub genes of the protein-protein interaction network.

**GeneID**	**ProteinID**	**Gene description**
Zm00001d045500	GRMZM2G048557_P01	39S ribosomal protein L47
Zm00001d013392	GRMZM2G046055_P01	probable histone H2AXa
Zm00001d005228	pco061453, pza02939	Ribosomal protein L22p/L17e-like
Zm00001d024373	GRMZM2G178807_P01	60S ribosomal protein L7-like
Zm00001d050375	csu614a	uncharacterized LOC100285226
Zm00001d045372	GRMZM2G050971_P01	iron-sulfur assembly protein IscA
Zm00001d014445	GRMZM5G845129_P01	protein ACTIVITY OF BC1 COMPLEX KINASE 7, chloroplastic
Zm00001d016561	pco145004	uncharacterized LOC100286235
Zm00001d014192	GRMZM2G120579_P01	uncharacterized LOC100276033
Zm00001d008244	GRMZM2G120857_P01	3-isopropylmalate dehydrogenase
Zm00001d042397	GRMZM2G432390_P01	ABC transporter B family member 26, chloroplastic
Zm00001d036738	GRMZM2G442804_P03	S-adenosyl-L-methionine-dependent methyltransferase superfamily protein

To further study the functions of leaf angle related genes, the 12 hub genes of this module were analyzed by GO enrichment and KEGG pathways. In GO analysis, the genes were mainly enriched in “Peptide metabolic process”, “rRNA binding”, “Structural constituent of ribosome”, “Structural molecule activity”, “Mitochondrial large ribosomal subunit”, “Mitochondrial ribosome”, “Plastoglobule”, “Large ribosomal subunit”, and “Mitochondrial matrix”. In KEGG pathway analysis, the hub genes were mainly involved in “Ribosome”.

## 4 Discussion

### 4.1 *ZmbHLH112* gene regulates maize leaf angle development

Leaf angle is an important agronomic trait in maize, with a smaller leaf angle allowing higher planting density, leading to more efficient light capture and higher yields (Tross et al., 2021). Cao et al. (Cao et al., 2020) cloned maize bHLH TF ZmIBH1-1 by map-based cloning, and found that it was a negative regulator of leaf angle. The combined transcriptomics results revealed 59 ZmIBH1-1-modulated target genes, which were mainly related to the cell wall, cell development, and hormones. Compared with the wild-type, the rice *lc2* (*rice leaf invocation2 (LC2*, three alleles)) mutant has a larger leaf angle due to increased division of paraxial epidermal cells in leaf joints. However, *LC2* is mainly expressed in the lamina joint during leaf development, and is particularly induced by the phytohormones abscisic acid, gibberellin acid, auxin, and brassinosteroids. The mutant lc2 had changed expression level of genes related to cell division and plant hormones due to the lack of the *LC2* gene, resulting in the change of leaf angle (Zhao et al., 2010). These results suggested that the smaller leaf angle of the mutant *bhlh112* compared with wild-type B73 may be caused by the lack of the *ZmbHLH112* gene. In order to verify this hypothesis, we used qRT-PCR to verify the relative expression of *ZmbHLH112*. The relative expression of this gene was the highest in the wild-type leaf angle, indicating that the gene has a regulatory effect on the development of the leaf angle in maize. Transcriptome analysis showed that auxin signal transduction, protein kinase activity, and membrane-related gene expression were down-regulated, which was consistent with the findings of previous studies, indicating that the smaller leaf angle of mutant *bhlh112* was also related to the differential expression of this gene.

### 4.2 Effect of exogenous IAA on maize leaf angle

The auxin indoleacetic acid (IAA) is critical in regulating the adaxial/abaxial cell growth of leaves in *Arabidopsis* (Ali et al., 2020); it also regulates the growth of *Arabidopsis* hypocotyls by affecting the expression of auxin response factor related genes (Reed et al., 2018). In the current study, the leaf angles of mutant *bhlh112* and wild-type B73 maize V3 stage reached the maximum under 100 µmol·L^-1^ exogenous IAA treatment. Transcriptome analysis showed that, compared with the control, the DEGs in the mutant *bhlh112* were involved in plant hormone signal transduction, flavonoid biosynthesis and some amino acid biosynthesis, with redox activity, hydrolase activity and transmembrane transporter activity, and were also cell membrane components. In addition, the DEGs in the wild-type participate in the cell membrane component, lipid metabolism process, flavonoid biosynthetic process and the composition of plasmodesmata and membrane, and have oxidoreductase activity and catalytic activity. Flavonoids are secondary metabolites, which play a variety of functions in plants and can regulate auxin transport (Mierziak et al., 2014). Our results showed that both the mutant and wild-type genes were involved in flavonoids biosynthesis, which indicated that exogenous IAA may cause an imbalance of endogenous auxin in plants, and flavonoids may be a regulator of auxin homeostasis in maize seedlings. At the same time, differential expression of genes related to cell development and plant hormone signal transduction caused the changes to maize leaf angle.

Glucosyltransferase *UGT74D1* affects leaf positioning through modulating auxin homeostasis and regulating transcription of related genes (Jin et al., 2021). *Rice LEAF INCLUSION1 (LC1)*, an IAA amide synthase, maintains auxin homeostasis by binding excess IAA with various amino acids, and then regulates the leaf angle (Zhao et al., 2013). Leaf invocation3 (LC3) containing the SPOC domain cooperatively regulates auxin signals by interacting with LIP1 (LC3-interacting protein 1, a HIT zinc finger domain-containing protein), and maintains auxin homeostasis to regulate lamina joint development in rice (Chen et al., 2018). These results indicate that maintenance of auxin homeostasis play an essential role in regulating leaf angle.

### 4.3 Effect of exogenous ABA on maize leaf angle

As an important plant hormone, abscisic acid (ABA) participates in the regulation of the physiological processes required to respond to various abiotic stresses in plants (Huang et al., 2017). ABA can regulate seed development, maturation, and dormancy (Verslues and Zhu, 2007). Under drought stress, ABA can induce stomatal closure and reduce water loss (Guajardo et al., 2016). It also has a defensive effect in plant immunity (Xie et al., 2018). However, there have been very few studies on ABA regulation of plant leaf angle. Li et al. found that ABA regulates the leaf angle of rice by the BR biosynthesis pathway and BR signal transduction (Li et al., 2019a). Here, we found that the leaf angle of mutant *bhlh112* and wild-type maize V3 stage reached the maximum under treatment with 10 µmol·L^-1^ exogenous ABA. Transcriptome analysis showed that, compared with the control, in the mutant *bhlh112*, the up-regulated DEGs mainly enriched the ribosome structural components, small ribosomal subunits, and membranes and membrane components; down-regulated DEGs mainly enriched plasmodesmata, auxin and abscisic acid signaling pathways, membrane transporter activity, and redox activity. In the wild-type, the up-regulated genes mainly enriched catalytic activity, serine pyruvate transaminase activity, glycine biosynthesis pathway and peroxisome, while the down-regulated genes enriched methyltransferase activity, hydrolase activity, lignin biosynthesis pathway, cell wall biogenesis, and cell wall components. These results suggest that ABA may regulate the development of leaf angle through the expression of these differentially expressed genes.

In addition, transcriptome analysis found that most of the differentially expressed genes were oxidoreductase, transferase, hydrolase and ribosome components; there were also genes involved in plant hormone signal transduction, such as *IAA24* (*Zm00001d018973*), a member of the *AUX/IAA* family, and genes related to cell wall structure, including *Zm00001d017033*, is a glycine-rich cell wall structural protein. Therefore, we speculate that ABA may regulate maize leaf angle through IAA signal transduction or BR biosynthesis (Li et al., 2019a).

### 4.4 *ZmbHLH112* modulates leaf angle through auxin and BR signaling

**Figure 11:**
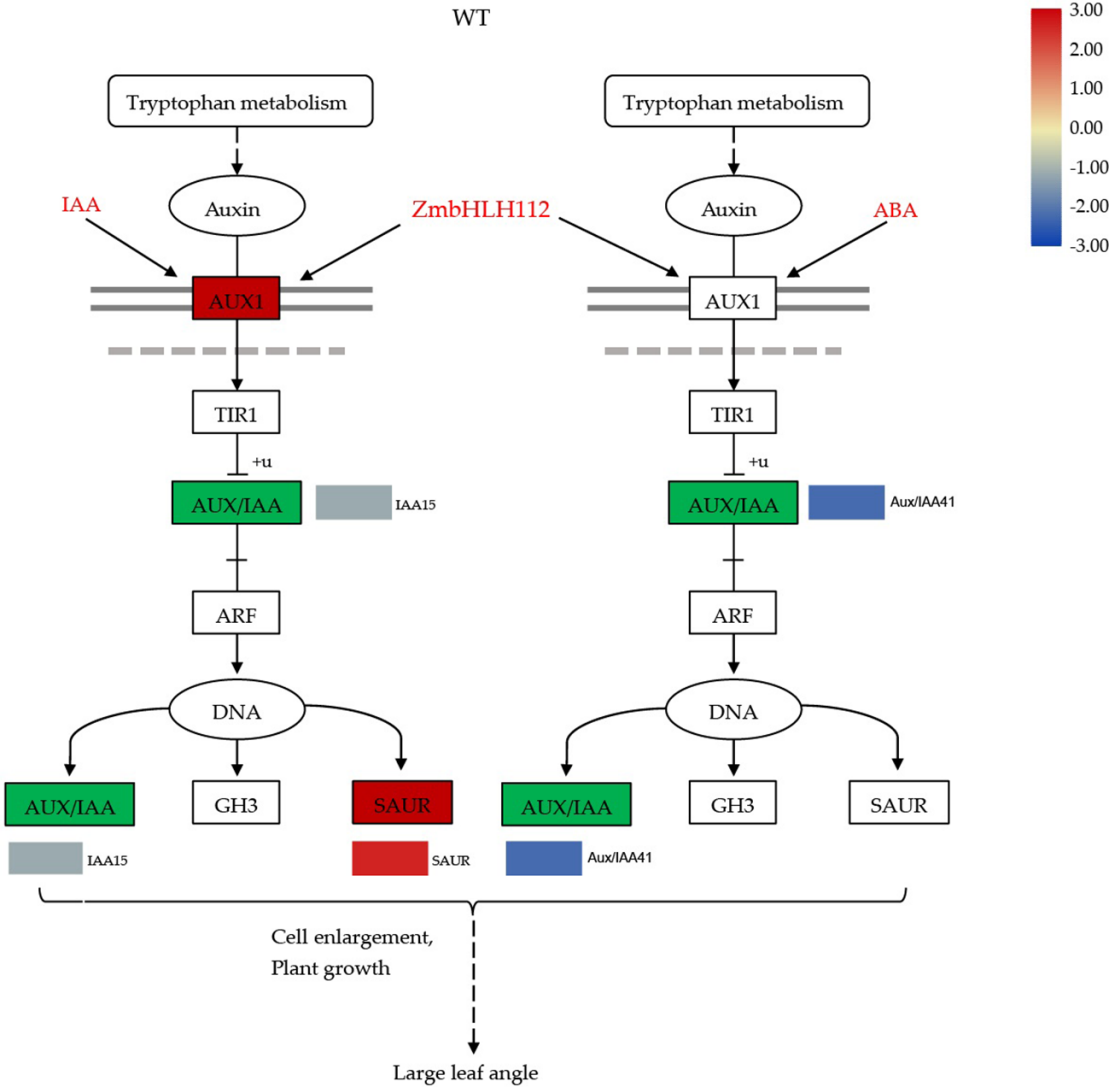
Molecular model for the regulation of maize leaf angle development by *ZmbHLH112*. *ZmbHLH112* mediates the auxin signaling pathway. WT: wild-type B73.

**Figure 12:**
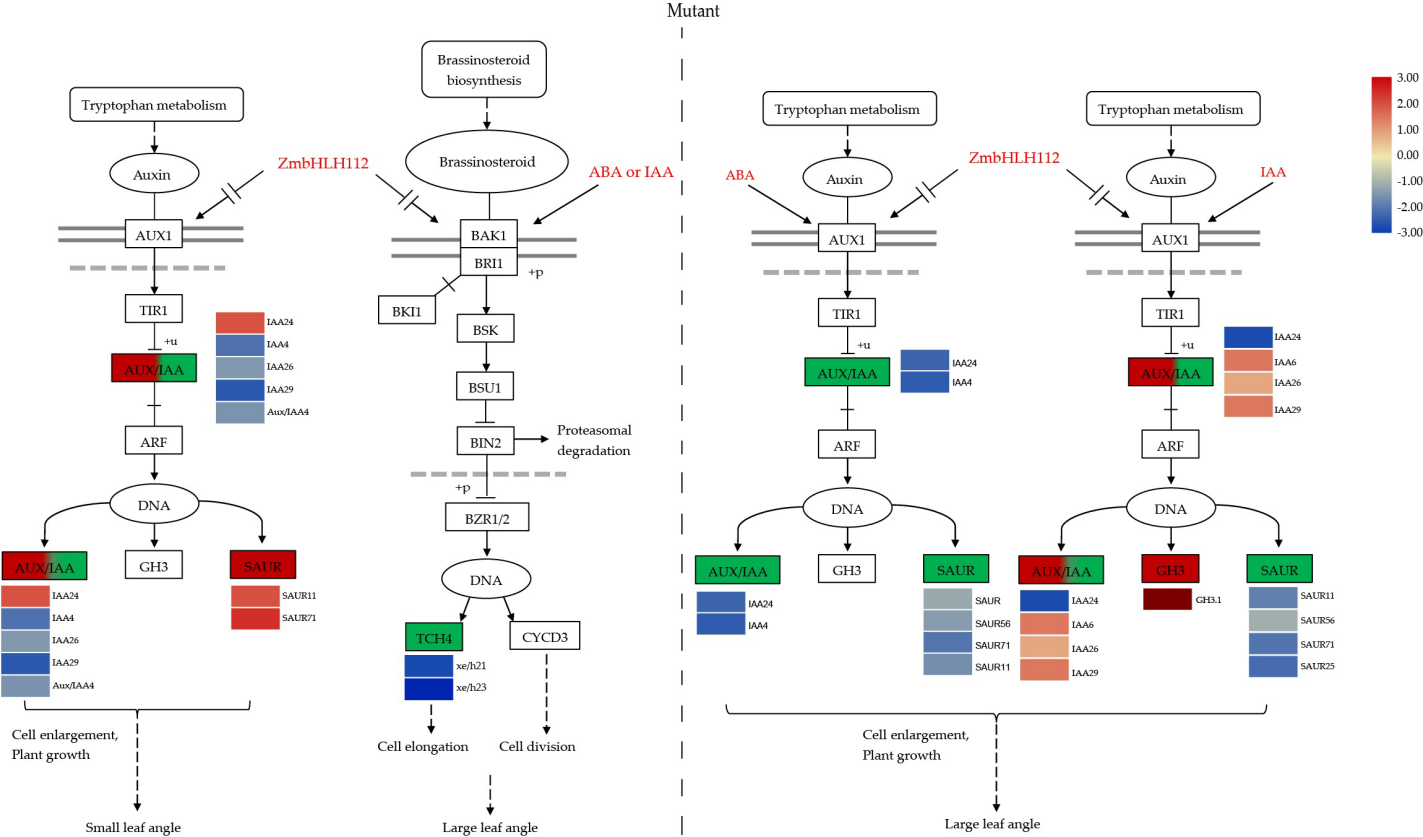
Two signaling pathways regulating maize leaf angle in the mutant *bhlh112*: the auxin signaling and BR biogenesis pathways. Mutant: *bhlh112*.

The development of leaf angle is affected by many factors, including the regulation of exogenous plant hormones and genes. Previous studies have shown that gene regulation occurs mainly through the auxin and brassinolide signal pathways, which then adjust the inclination of the leaf angle (Liu et al., 2019; Tian et al., 2021). The bHLH TFs play a variety of regulatory roles in plant growth and development. Dong et al. (Dong et al., 2018) found that 6 members of the *bHLH* family share a conserved function in regulating the flag leaf angle in rice. This study found that the *ZmbHLH112* gene can repress the expression of *Aux/IAA* related genes (auxin/indole-3-acid acid transcription repressors), promote the binding of auxin response factor (ARF) and DNA, and then regulate the elongation of the leaf angle cells. Aux/IAA and ARF the central components of the auxin signaling pathway and play an important role in auxin mediated growth and development (Liscum and Reed, 2002). Aux/IAA, Gretchen Hagen3 (GH3), and small auxin-up RNA (SAUR) are three types of auxin response genes. SAUR can be rapidly induced by exogenous auxin to promote plant growth and development. We found that exogenous IAA or ABA treatment caused the differential expression of SAUR (**Figures 11**, **12**) (Li et al., 2017; Zhang et al., 2021b). The GH3 protein catalyzes conjugation of amino acids with free IAA, jasmonic acid (JA), and salicylic acid (SA), and auxin response elements (AuxRE) exist in its promoter region. The binding of auxin response factors (ARFs) with AuxRE can regulate the expression of GH3 related genes, regulate auxin homeostasis in plants, and affect the development of the leaf angle (Yu et al., 2018). In addition, we found that in the mutant *bhlh112*, exogenous IAA and ABA treatment caused down-regulation of *TCH4* related genes in the BR biosynthesis pathway; Xyloglucan endoglucokinase/hydrolase (XTH) encoded by the *TCH4* gene is the primary component that acts on the cell wall and plays an essential role in plant morphogenesis (Iliev et al., 2002; Zhang et al., 2022). In conclusion, this study revealed that the *ZmbHLH112* gene of the bHLH transcription factor family acts on genes related to cell elongation and cell wall formation in the leaf angle of maize, and regulates the inclination of the leaf angle through the auxin signaling and BR biosynthesis pathways.

### 4.5 Co-expression network in maize leaf angle

By comparing RNA-seq with maize seeding leaf angle traits, 12 hub genes were identified. The GO enrichment KEGG pathway analysis of these genes showed that most genes had ribosome structure molecular activity, rRNA binding activity, or mitochondrial large ribosomal subunit activity. We speculate that these hub genes may play a role in regulating maize leaf angle through modifying some characteristics of ribosomes. The significant expression of ribosomal subunit related genes was also found when application of exogenous ABA regulated the leaf angle of mutant *bhlh112*, which further indicated that ribosomal subunits may play an important role in leaf development.

The rice gene *RML1* encodes a ribosomal large subunit protein 3B (RPL3B). The free 60s ribosomes and polysomes in the functional defective mutant *rice minute-like1* (*rml1*) were significantly reduced, resulting in plant phenotypic diminution, narrow leaves, and growth retardation. Those results showed that the ribosomal protein RPL3B is required for maintaining normal leaf morphology and plant architecture in rice through its regulation of ribosome biogenesis (Zheng et al., 2016). *Arabidopsis* rRNA methyltransferase CMAL (Chloroplast MraW-Like) is involved in plant development, likely by modulating auxin derived signaling pathways. Knockout of the *CMAL* gene leads to chloroplast functional defects, as well as abnormalities in leaf and root development and in the overall plant morphology (Zou et al., 2020). Kojima et al. identified a new nucleolar protein G-patch domain protein1 (GDP1) in *Arabidopsis*, which is involved in the ribosome biosynthesis pathway. GDP1 functional deletion mutation causes a proliferation defect in leaf primordium cells, and in the adaxial - abaxial polarity regulation of leaves (Kojima et al., 2017). These results showed that the large ribosomal subunit could regulate the development of plant leaves; however, the molecular mechanism of leaf angle adjustment needs further study.

## 5 Conclusions

In this study, the wild-type B73 produced the mutant *bhlh112* by EMS-induction. Our results showed that the 1241st base G of the *ZmbHLH112* gene in the mutant was mutated into A, whereby tryptophan was mutated into termination codon. Compared with the wild-type, the mutant *bhlh112* had lower plant height and smaller leaf angles. Wild-type and mutant seedlings were treated with exogenous IAA and ABA to screen the optimal concentrations of each, and the functions of *ZmbHLH112* were analyzed by transcriptome sequencing. The results showed that *ZmbHLH112* is related to auxin signal transduction and BR biosynthesis, and involved in the activation of auxin and brassinolide signal pathway related genes and cell growth. Further transcriptome analysis showed that genes involved in cell wall, cell membrane development, plant hormone signal transduction, ribosome biosynthesis, and flavonoid biosynthesis were differentially expressed between the mutant *bhlh112* and wild-type B73, resulting in the change in leaf angles in the mutant. The co-expression network was constructed by WGCNA, and 12 hub genes related to the development of leaf angle were screened; In GO enrichment and KEGG pathways, the genes were mainly enriched in rRNA binding, ribosome biogenesis, Structural constituent of ribosome; Result showed that ribosomal subunits may play an important role in leaf development. This study provides an important finding allowing further elucidation of the molecular mechanism of regulation of leaf angle in maize.

## Data availability statement

The data presented in the study are deposited in the SRA at NCBI repository, accession number PRJNA856349. https://dataview.ncbi.nlm.nih.gov/object/PRJNA856349?reviewer=7t6aa2bj0no68d49hjr5bbbkpv.

## Author contributions

YP designed the experiments. YZ wrote the manuscript. YZ performed the experiments and analyzed data. XJ, JX, and YW participated in the planting and identification of experimental materials and the critical reading and discussion of the manuscript. All authors contributed to the article and approved the submitted version.

## Funding

This research was supported by the industrial support plan for colleges and universities of Gansu, China (No. 2022CYZC-46), the Fuxi Talent Project of Gansu Agricultural University, China (No. GAUFX-02Y09), and the Lanzhou Science and Technology Bureau (No. 2020-RC-122).

## Acknowledgments

We thank the reviewers for the critical review of the manuscripts, and Peng Yunling for her guidance and revision.

## Conflict of interest

The authors declare that the research was conducted in the absence of any commercial or financial relationships that could be construed as a potential conflict of interest.

## Publisher’s note

All claims expressed in this article are solely those of the authors and do not necessarily represent those of their affiliated organizations, or those of the publisher, the editors and the reviewers. Any product that may be evaluated in this article, or claim that may be made by its manufacturer, is not guaranteed or endorsed by the publisher.
