# Corrigendum: Morphological characterization and transcriptome analysis of leaf angle mutant *bhlh112* in maize [*Zea mays* L.]

**DOI:** 10.3389/fpls.2022.1089402

**Published:** 2022-11-24

**Authors:** Yunfang Zhang, Xiangzhuo Ji, Jinhong Xian, Yinxia Wang, Yunling Peng

**Affiliations:** ^1^ College of Agronomy, Gansu Agricultural University, Lanzhou, China; ^2^ Gansu Provincial Key Laboratory of Aridland Crop Science, Gansu Agricultural University, Lanzhou, China; ^3^ Gansu Key Laboratory of Crop Improvement & Germplasm Enhancement, Gansu Agricultural University, Lanzhou, China

**Keywords:** maize (*Zea mays* L.), *bhlh112* mutant, transcriptomics, exogenous hormones, co-expression network

In the published article, there were 2 errors. A sentence in the **Conclusions** of the article was not revised in time, resulting in a difference from the analysis of the results in the text. This sentence previously stated:

“Our results showed that the 1241st base G of the *ZmbHLH112* gene in the mutant was mutated into A, and glycine was mutated into aspartic acid.”

The corrected sentence appears below:

“Our results showed that the 1241st base G of the *ZmbHLH112* gene in the mutant was mutated into A, whereby tryptophan was mutated into termination codon.”

There was also an error in the caption of **Figure 2** . This sentence previously stated:

“(E) Corresponding leaf angle in (D, F) Mature ear of wild type and mutant.”

The corrected sentence appears below:

“(E) Corresponding leaf angle in D; (F) Mature ear of wild type and mutant.”

The authors apologize for these errors and state that this does not change the scientific conclusions of the article in any way. The original article has been updated.

## Publisher’s note

All claims expressed in this article are solely those of the authors and do not necessarily represent those of their affiliated organizations, or those of the publisher, the editors and the reviewers. Any product that may be evaluated in this article, or claim that may be made by its manufacturer, is not guaranteed or endorsed by the publisher.

